# Multiple Genes Cause Postmating Prezygotic Reproductive Isolation in the *Drosophila virilis* Group

**DOI:** 10.1534/g3.116.033340

**Published:** 2016-10-10

**Authors:** Yasir H. Ahmed-Braimah

**Affiliations:** Department of Biology, University of Rochester, New York 14627

**Keywords:** *Drosophila*, reproductive isolation, gametic incompatibility, genetics of speciation

## Abstract

Understanding the genetic basis of speciation is a central problem in evolutionary biology. Studies of reproductive isolation have provided several insights into the genetic causes of speciation, especially in taxa that lend themselves to detailed genetic scrutiny. Reproductive barriers have usually been divided into those that occur before zygote formation (prezygotic) and after (postzygotic), with the latter receiving a great deal of attention over several decades. Reproductive barriers that occur after mating but before zygote formation [postmating prezygotic (PMPZ)] are especially understudied at the genetic level. Here, I present a phenotypic and genetic analysis of a PMPZ reproductive barrier between two species of the *Drosophila virilis* group: *D. americana* and *D. virilis*. This species pair shows strong PMPZ isolation, especially when *D. americana* males mate with *D. virilis* females: ∼99% of eggs laid after these heterospecific copulations are not fertilized. Previous work has shown that the paternal loci contributing to this incompatibility reside on two chromosomes, one of which (chromosome 5) likely carries multiple factors. The other (chromosome 2) is fixed for a paracentric inversion that encompasses nearly half the chromosome. Here, I present two results. First, I show that PMPZ in this species cross is largely due to defective sperm storage in heterospecific copulations. Second, using advanced intercross and backcross mapping approaches, I identify genomic regions that carry genes capable of rescuing heterospecific fertilization. I conclude that paternal incompatibility between *D. americana* males and *D. virilis* females is underlain by four or more genes on chromosomes 2 and 5.

Genetic studies of reproductive isolation have provided important insights into speciation (Coyne and Orr 2004). For example, many of the identified genes that cause postzygotic isolation in *Drosophila* show a signature of rapid adaptive change (Ting *et al.* 1998; Barbash *et al.* 2003; Presgraves *et al.* 2003; Phadnis and Orr 2009). Moreover, the genes that are known to cause postzygotic isolation have a range of functions, suggesting that no particular biological process underlies these barriers (Orr 2005). These insights followed from the identification of a set of specific genes that cause reproductive isolation between species. Similar progress has not, however, been made for prezygotic reproductive isolation in *Drosophila*. This is unfortunate, as we know that prezygotic isolation is at least as important as postzygotic isolation. Indeed, a survey of many *Drosophila* species pairs shows that pre- and post-zygotic reproductive isolation evolve at roughly similar rates in allopatry, but that the former evolves faster than the latter in sympatry (Coyne and Orr 1989, 1997). The same questions that geneticists asked about postzygotic barriers can be asked about prezygotic ones: how many genes cause prezygotic isolation between species? What are the identities of these genes? What biological functions are affected in species isolated by prezygotic barriers? Finally, what evolutionary forces drive divergence of the genes that cause prezygotic isolation? While studies in *Drosophila* have begun to provide answers to some of these questions (reviewed in Laturney and Moehring 2012), a great deal remains unclear.

Much work has focused on the biological functions that are likely affected in prezygotic barriers, as well as the evolutionary forces that likely bring them about (Ritchie 2007; Panhuis *et al.* 2001). In particular, sexual selection is thought to be a major force that differentiates sexual characters—those involved in mate-recognition, courtship, and/or fertilization—between allopatric populations. Theoretical work has shown that sexual selection can create a genetic correlation between male and female sexual characters within a population (Lande 1981; Kirkpatrick 1982), which can cause divergent populations to suffer lowered sexual compatibility. Initially proposed by Fisher (1932), this “run-away” process might, in principle, be a potent force in speciation. Similarly, other forms of sexual selection, including intra- and inter-sexual conflict, might also drive rapid evolutionary change, especially in components of the reproductive system (Rice 1998; Gavrilets 2000). Prezygotic barriers clearly, then, might result from sexual selection within geographically isolated species/populations. A genetic understanding of the link between sexual selection and speciation, however, has remained elusive (Ritchie 2007).

Some forms of prezygotic barriers between species act after mating but before the formation of the zygote. These barriers, so-called postmating prezygotic (PMPZ) barriers, generally involve an incompatibility between the male ejaculate and the female reproductive tract. Such barriers can arise as a consequence of sexual selection as described above. The fact that sexual selection can take place after mating (postcopulatory sexual selection) is now well recognized (Parker 1970; Eberhard 1996). Phenotypes that rapidly evolve due to these forces can underlie PMPZ reproductive isolation across a wide range of animal and plant taxa (Howard *et al.* 2009; Moyle *et al.* 2014).

PMPZ reproductive barriers hold particular promise in addressing some of the shortcomings in the genetic study of prezygotic isolation highlighted above. For instance, the male component of PMPZ isolation represents a fairly well characterized portion of the genome, *i.e.*, genes whose products reside in the ejaculate. The presumed targets of postcopulatory sexual selection in males obviously include, but are not limited to, sperm and the accompanying seminal fluid components. Those targets are easily identifiable across a wide range of taxa using modern “omics” tools (Findlay *et al.* 2009; Wasbrough *et al.* 2010; Dean *et al.* 2011; Sirot *et al.* 2011). Furthermore, analysis of reproductive genes over the past two decades has highlighted a striking pattern: these proteins tend to evolve rapidly by positive selection between closely related species (Swanson and Vacquier 2002; Haerty *et al.* 2007). Such a pattern suggests that the potential targets of postcopulatory sexual selection within the male ejaculate can also be identified using standard molecular population genetics methods.

The *virilis* subgroup of *Drosophila* represents a nearly ideal system for the genetic study of PMPZ barriers in insects. PMPZ isolation is prevalent among members of this group, which includes *D. americana*, *D. novamexicana*, *D. lummei*, and *D. virilis*, and other barriers are less common (Sagga and Civetta 2011; Sweigart 2010b). PMPZ is also observed between allopatric populations of *D. montana* (Jennings *et al.* 2014), an outgroup species that is a member of the larger *virilis* clade, highlighting the rapidity with which PMPZ can evolve. Indeed, in the most closely related pair of species (*D. americana* and *D. novamexicana*, diverged ∼0.5 MYA), PMPZ isolation is the only reproductive barrier observed in the laboratory (Ahmed-Braimah and McAllister 2012). Further, with the exception of the cross between *D. virilis* and *D. lummei*, all pair-wise crosses between members of this group result in a ≥98% reduction in egg hatchability after heterospecific copulations in at least one direction of the cross (Sagga and Civetta 2011). For example, *D. americana* males fertilize ≤2% of *D. novamexicana*, *D. lummei*, and *D. virilis* eggs. *D. americana* females, on the other hand, are somewhat more compatible with *D. novamexicana* and *D. virilis* males (20–30% hatch rate) but are highly incompatible with *D. lummei* males (1% hatch rate) (Sweigart 2010b; Sagga and Civetta 2011; Ahmed-Braimah and McAllister 2012). The reduced hatchability in the *D. americana*–*D. novamexicana* and *D. novamexicana*–*D. virilis* crosses has been shown to result from failure of heterospecific sperm to fertilize the egg, possibly due to improper sperm storage, incapacitation, or ejection (Sagga and Civetta 2011; Ahmed-Braimah and McAllister 2012). Thus, several pairs of species in the *virilis* group are well suited for the genetic study of PMPZ.

The PMPZ incompatibility between *D. virilis* females and *D. americana* males obviously must involve paternal genes in *D. americana*. These genes either indirectly affect ejaculate characteristics or their protein products are themselves components of the ejaculate. A previous genetic analysis showed that these paternal factors act recessively between species and map to two chromosomes, 2 and 5 (Sweigart 2010b). Chromosome 2 carries a fixed inversion difference between *D. americana* and *D. virilis* that affects the centromeric half of the chromosome. Chromosome 5 is largely homosequential between the two species, although some *D. americana* strains carry a segregating inversion on that chromosome (Hsu 1952)). In a previous study by Sweigart (2010b), the mapping approach used recombinant first-generation backcross individuals that were either homozygous for *D. americana* alleles or heterozygous. Individuals heterozygous at the relevant PMPZ loci have increased fertilization success relative to pure *D. americana* when mated with *D. virilis* females. Three paternal quantitative trait loci (QTL) were identified on the two chromosomes. A single QTL mapped to the inverted region on chromosome 2 and two adjacent QTL map to a 7.3 Mb region on chromosome 5. The fixed inversion difference on chromosome 2 suppresses recombination in heterozygotes, precluding further fine mapping of this paternal QTL. Chromosome 5, however, recombines in heterozygotes, allowing fine-mapping of the paternal factor(s) on that chromosome.

Here, I examined two aspects of PMPZ isolation between *D. americana* males and *D. virilis* females. First, I analyzed the phenotypic basis of the PMPZ incompatibility. In particular, I analyzed the dynamics of sperm storage after heterospecific copulations compared to conspecific ones across multiple time points. Previous work has shown that ∼99% of eggs laid after heterospecific copulations are not fertilized (Sweigart 2010b). It is unknown, however, whether sperm are transferred and stored successfully in these crosses.

Second, I extended the genetic analysis performed previously by fine-mapping regions on chromosome 5 that are involved in PMPZ isolation. To accomplish this, I used a three-step approach. First, I used a visible-assisted QTL mapping approach on an advanced recombinant backcross population. Second, I generated stable lines that carry recombinant chromosomes that have been introgressed into a *D. americana* genetic background. Finally, I used two of these recombinant introgression lines (RecIntLs) to further recombine the fifth chromosome and further fine-map the paternal loci. Ultimately, the goal is to identify regions on chromosome 5 that, when heterozygous in a hybrid male, rescue fertilization when that male is crossed with *D. virilis* females.

In the phenotypic analysis I conclude that the PMPZ phenotype involves rapid loss of heterospecific sperm from female storage within several hours after copulation. Sperm loss is most pronounced from the seminal receptacle. In the genetic analysis I confirm that at least two paternal factors are present on the middle of chromosome 5, but that an additional significant QTL near the centromere is also involved. Ultimately, I identify a ∼4 Mb region in the middle of chromosome 5 that, by itself, has a modest effect on fertilization success but has a major effect in conjunction with the adjacent and centromeric factors.

## Materials and Methods

### Fly strains and husbandry

Flies were maintained at a constant temperature (22°), on a ∼12-hr day/night cycle, and were fed standard cornmeal medium. The *D. virilis* strain used in all crosses was created by crossing two strains, 10510-1051.31 and 10510-1051.55, both of which were obtained from the University of California San Diego *Drosophila* Species Stock Center (stockcenter.ucsd.edu). Strain 10510-1051.31 carries two visible mutations on the fifth chromosome: Branched (*B*) and scarlet (*st*). Strain 10510-1051.55 carries another visible mutation, varnished (*va*), on the second chromosome. All three visible mutations behave recessively in a *D. americana* genetic background. Both *B* and *va* were made homozygous in the new *D. virilis* strain used throughout this study, but *st* remained segregating. *D. virilis* is also distinguishable from *D. americana* during the pupal stage, where *D. virilis* features black pupae and *D. americana* features brown pupae. A single gene causes this color difference and its exact location and identity are known (Ahmed-Braimah and Sweigart 2015). Pupal case color was, therefore, used as an additional visible marker on chromosome 5 (hereafter abbreviated as “*pup*”). The *D. americana* strain used in all crosses (SB02.06) was provided by Dr. Bryant F. McAllister (University of Iowa). This strain is homosequential with *D. virilis* along most of chromosome 5, but contains a large inversion that is fixed between *D. americana* and *D. virilis* along the centromeric half of chromosome 2. For all crosses (unless otherwise noted), male and female flies were collected within 2 d of eclosion and reared separately until sexually mature and crossed at 12–14 d. To obtain progeny counts, mating pairs were housed for ∼10 d, after which males were removed from the vials and females were allowed to lay eggs. Progeny counts were obtained in several bouts ≥3 wk later.

### Sperm storage dynamics after conspecific and heterospecific matings

To analyze sperm storage dynamics in conspecific and heterospecific matings, virgin males from both species and virgin *D. virilis* females were collected as described above. Individual virgin females were housed with a single conspecific or heterospecific male (without anesthesia) until copulation occurred. After copulation, males were removed from the vial, and each inseminated female was assigned to one of six dissection times: 1, 3, 6, 12, 24, and 144 hours post copulation (*n* ∼ 10 females per cross-type and time-point). Each vial was assigned an ID such that, during dissections, the cross-type is not evident. During dissections, the female reproductive tract (not including the ovaries) was extracted and mounted onto a glass slide with 1× PBS solution and a cover slip. The reproductive tract was observed under a dark field light microscope (Nikon Optiphot-2) and the presence/absence of sperm (irrespective of motility) was scored and images/videos were captured. Importantly, each female’s sperm storage status was assigned without knowledge of cross-type. Presence or absence of sperm was scored by eye as “many,” “few,” “very few,” or “none.” A $\chi^{2}$ contingency test was used to examine significant differences between conspecific and heterospecific matings in the first 24 hr.

### Visible-assisted recombinant mapping of paternal PMPZ loci

Mapping the paternal factors on chromosome 5 was carried out in three steps. First, I performed a comprehensive QTL analysis using advanced-generation backcross individuals that were selected based on known recombination events between two visible markers on chromosome 5. Second, I generated lines that carry recombinant fifth chromosomes introgressed into a *D. americana* background. Finally, I used two of the RecIntLs to further recombine the fifth chromosome. I describe each of these steps in further detail below.

#### QTL analysis in advanced-recombinant backcross individuals:

The recombinant mapping population was generated as follows (crossing scheme shown in Figure 2). Large numbers of F3 and F4 advanced hybrid progeny were generated from a cross of parental *D. americana* females and *D. virilis* males. To increase mapping power, F3 and F4 hybrid males were genotyped at two visible markers on chromosome 5, *B* and *pup*. All males selected carry at least one recombinant chromosome between the two visible markers. These males were then individually crossed with *D. americana* females and subsequently frozen at −20° for molecular genotyping. Sires from these males (up to 20 per recombinant father) were used in single matings with *D. virilis* females to assess the phenotypic effect of the recombinant chromosome they carry (*i.e.*, recombinant chromosomes were tested in replicate). Sires of each recombinant male were also frozen for molecular genotyping. Ultimately the mapping population consisted of 1842 individuals, sired by 220 recombinant males. Genotyping of microsatellite markers was carried out as described previously (Sweigart 2010b; Ahmed-Braimah and Sweigart 2015). A total of 38 microsatellite markers (34 on chromosome 5 and four on chromosome 2) were used to genotype recombinant individuals (Supplemental Material, Table S1).

QTL for high progeny production in this population were mapped using a series of approaches implemented in the R/qtl package (Broman *et al.* 2003). First, a single-QTL genome scan was performed using the “scanone” function [this and subsequent analyses were run using Haley–Knott (HK) regression (Haley and Knott 1992)]. Second, a two-QTL scan was performed using the “scantwo” function. Third, a set of multiple QTL models (MQM) were examined, taking into account the putative QTL identified in the two previous scans, and their effect sizes and potential epistatic interactions were evaluated. Finally, an implementation of composite interval mapping (CIM) in R/qtl was used to examine QTL intervals and to compare the results to the best fit MQM model. CIM was run by varying the number of marker covariates, which are forward selected and correspond to putative QTL locations. The genetic map used in all QTL analyses was estimated from the data, and thus shows inflated distances near the visible markers between which recombinants were selected (Figure S1). Significance thresholds in all cases were estimated by permutation (*n* = 1000). The genotype/phenotype data, R code, and accompanying files used to perform the QTL analysis are provided in File S1, File S2, File S3, and File S4.

#### Generating RecIntLs:

The fifth chromosomes of a set of recombinant males (*n* = 43) generated above were introgressed into a *D. americana* genetic background by backcrossing carrier males for 3–5 generations. Meiotic recombination does not occur in *Drosophila* males, therefore the recombinant fifth chromosome remains intact every generation. Each generation, inheritance of the recombinant fifth chromosome was ensured by genotyping at diagnostic microsatellite markers. After repeated backcrossing, introgression heterozygotes were mated to each other and only progeny that were homozygous for the corresponding visible marker (*pup* or *B*) were collected to initiate the respective RecIntLs. It’s important to note that the introgressed material here is the entire recombinant chromosome, whose alleles differ between each RecIntLs, and may vary slightly within each RecIntL due to possible recombination events when making the line homozygous for the recombinant fifth chromosome. The reproductive success of RecIntL males was assessed by individually crossing a subset of males from each line (∼10–50) to *D. virilis* females and counting their progeny, as described above.

#### Further recombination of two RecIntL lines:

Two of the RecIntLs (F3BR-59-B and F4BR-96) were chosen to generate further recombinants and to allow finer mapping on chromosome 5 (crossing scheme shown in Figure S2). Males from the two lines (∼20) were crossed with a large set (∼100) of *D. americana* females. Their offspring were allowed to intercross for two generations to allow additional recombination. After two generations, flies were screened for the presence of the visible mutation—*B* in both cases. Males homozygous at *B* were then mated to *D. americana* females to produce sets of brothers that were heterozygous for either a parental or a recombinant fifth chromosome. Newly recombined fifth chromosome males were phenotyped by crossing with *D. virilis* females as described above. The level of recombination was assessed among newly generated *B* homozygotes and their male sires by genotyping along the heterozygous region in the original RecIntL.

### Data availability

The strains generated here are available upon request. File S1 contains code used to perform the QTL analysis and generate figures. File S2 contains the phenotype and genotype data. File S3 contains chromosome 5 physical map distances. File S4 contains the inferred QTL intervals and log_10_ likelihood (LOD) scores.

## Results

### The phenotypic basis of PMPZ isolation between D. americana and D. virilis

PMPZ isolation between *D. americana* males and *D. virilis* females is characterized by a dramatic reduction in fertilization rates in heterospecific copulations relative to conspecific ones (Sweigart 2010b). However, the functional basis of this incompatibility is not well understood. In *Drosophila*, females store sperm for extended periods of time in two sperm storage organs: the seminal receptacle and a pair of spermathecae. Mated *D. virilis* females also dramatically increase their egg-laying rate within 24 hr after copulation. The events that go awry during PMPZ must occur during this period, either due to sperm incapacitation, loss, inability of stored sperm to enter the ova, or a combination of such problems.

**Figure 1:**
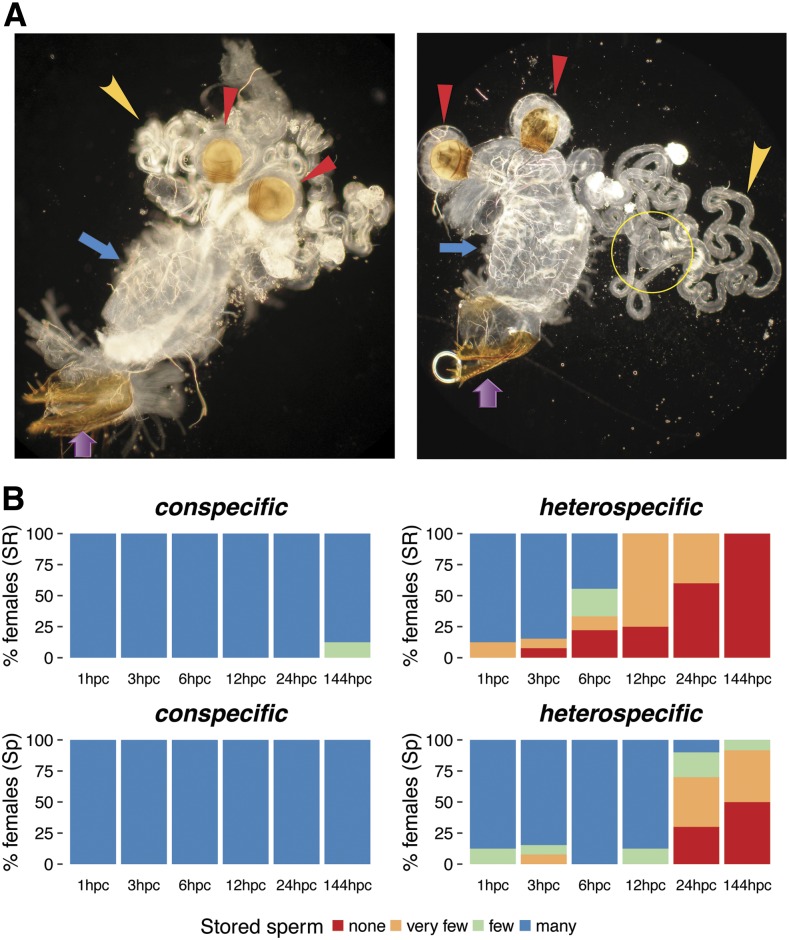
Sperm storage dynamics in conspecifically and heterospecifically inseminated *D. virilis* females. (A) *D. virilis* female reproductive tract 24 hr after conspecific (left) and heterospecific (right) insemination. Purple and blue arrows indicate the vagina and bursa, respectively. Red arrowheads indicate the paired spermathecae, and yellow arrowheads point to the long seminal receptacle. Sperm can be seen on the left panel as white material in the seminal receptacle, the spermathecal ducts, and lower portion of the bursa (File S5 shows a movie of this sample). The yellow circle on the right panel highlights the magnified region in File S6. (B) Bar graphs of percentage of females that contain none, very few, few, or many sperm at six time-points after conspecific and heterospecific copulation. Percentages are shown for the seminal receptacle (SR; top) and spermathecae (Sp; bottom). hpc, hours post copulation.

To explore these possibilities I examined the dynamics of sperm storage after conspecific and heterospecific inseminations across multiple time points in the first 24 hr after copulation. I also examined sperm storage 6 d after copulation. Using a dark-field light microscope with 100–400× magnification, sperm can easily be observed within the female reproductive tract. Sperm is also found densely packed in the spermathecae of females that are inseminated conspecifically. In the seminal receptacle, sperm appear white and highly motile. Males typically transfer more sperm than females can store, thus females eject a significant number of sperm that can sometimes be observed in the bursa near the vaginal opening (Figure 1A).

Among all conspecifically inseminated females, sperm is stored within 1 hr after mating in both storage organs, and the sperm appear highly mobile (Figure 1B). Indeed, sperm remain in storage for several days. In heterospecifically inseminated females, on the other hand, sperm enter and remain in storage for 2–3 hr after mating, but are gradually lost thereafter, especially from the seminal receptacle ($\chi^{2}=37.4,$ *d.f*. = 3, *p* value = 3.67e-08; Figure 1B). In particular, by 6 hr after mating, ∼50% of heterospecifically inseminated females contain far fewer sperm in the seminal receptacle than conspecifically inseminated females. By 24 hr, ∼60% of heterospecifically inseminated females contain almost no sperm in the seminal receptacle. In contrast, loss of sperm from the spermathecae is slower among heterospecifically inseminated females ($\chi^{2}=15.9,$ *d.f*. = 3, *p* value = 0.001; Figure 1B); nearly half these females contain some sperm in that organ even after 6 d.

These results show that heterospecific sperm successfully enters storage immediately after mating, but are rapidly ejected by *D. virilis* females, especially from the seminal receptacle. These observations provide the most likely biological basis of PMPZ isolation, but do not rule out additional incompatibilities that may occur before, or at the onset of fertilization.

### Mapping the paternal PMPZ loci

Previous work showed that the male genes in *D. americana* that are incompatible with the *D. virilis* female reproductive tract act recessively. Put differently, individuals that are heterozygous for these factors between species have increased fertilization success relative to individuals that are homozygous for *D. americana* alleles (Sweigart 2010b). Furthermore, genetic mapping showed that these loci reside on chromosomes 2 and 5. Here, I focus on mapping the paternal loci that reside on chromosome 5. Previously, this chromosome was found to contain two adjacent, major-effect QTL near the center of the chromosome that span 7.3 Mb (Sweigart 2010b). In this paper, I improve the mapping resolution of the loci within this region and elsewhere on the chromosome.

#### QTL analysis:

**Figure 2:**
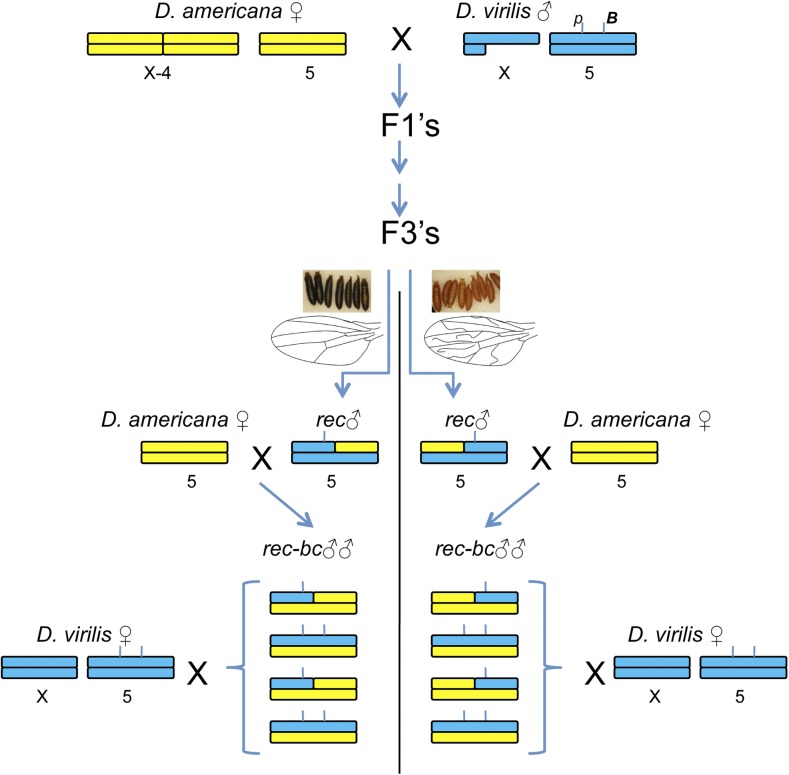
Crossing scheme to generate advanced-generation backcross males for the QTL analysis. Chromosome regions of *D. americana* and *D. virilis* are shown in yellow and blue, respectively. The approximate position of the two visible markers (*p* and *B*) used to select recombinants is indicated in the *D. virilis* male karyotype. The selection of recombinant individuals is shown here at the F3 generation, and example single-recombinant males are depicted. *rec*, recombinant; *rec-bc*, recombinant backcross.

Advanced intercross hybrids may contain chromosomes that have undergone multiple rounds of recombination. To select for recombinants around the candidate QTL region on chromosome 5, I used two visible genetic markers near the center of the chromosome (*B* and *pup*) and screened advanced intercross individuals (F3’s or F4’s) for recombination between these markers (Figure 2). Recombinant males were then individually crossed with *D. americana* to create the sibling pool that was heterozygous for at least one recombinant chromosome between the two visible markers. Importantly, this approach allows examination of replicate recombinant chromosomes. I tested the fertilization ability of these recombinant chromosomes by individually crossing recombinant males with *D. virilis* females and counting their progeny (Figure 2).

**Figure 3:**
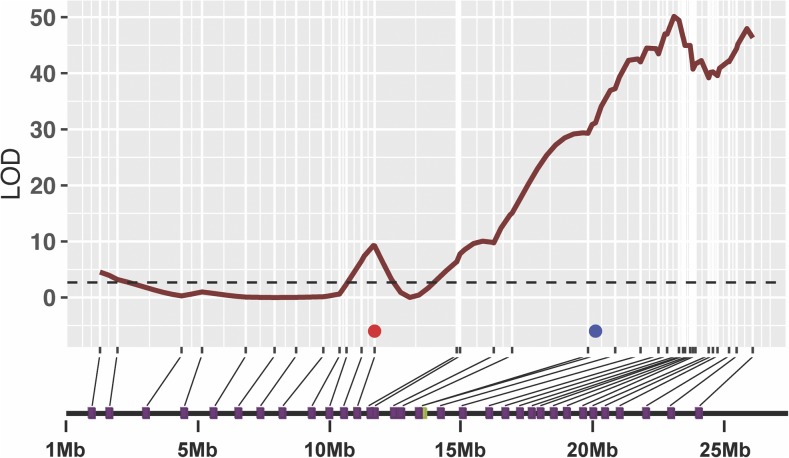
Single-QTL LOD profile of advanced-generation backcross individuals on chromosome 5. The significance threshold for the genome-wide scan is depicted with a dashed line (α=0.01). LOD scores are plotted against the genetic map distance for each microsatellite marker. Marker positions on the physical map (purple squares) are shown below the genetic map. The position of the two visible markers used to enrich for recombination are shown in red (*pup*) and blue (*B*). The centromere in this figure and all subsequent figures is on the right.

As a first step in the QTL analysis, I performed a single-QTL scan on chromosomes 2 and 5. Here, a model of a single QTL within a chromosome is compared to a model with no QTL on that chromosome. This analysis confirmed the previous results of Sweigart (2010b). Specifically, a significant QTL mapping to the inversion on chromosome 2 was seen (Figure S3A), as were two significant QTL on chromosome 5 (Figure 3 and Figure S3A). The right-most QTL on chromosome 5, however, extends into the centromeric region of the chromosome and spans more than half the length of the chromosome. The peak with the highest LOD score on chromosome 5 converges precisely on the same microsatellite marker (SSR116) identified by Sweigart (2010b), and has the largest effect on the phenotype. Under the current model, an individual with a heterozygous genotype at this marker increases progeny production by 24.04 relative to an individual that is homozygous for *D. americana* alleles at that marker (Figure S3B). In contrast, heterozygosity at the chromosome 2 QTL (at the visible marker *va*) increases progeny production by 19.69. The joint action of these two QTL under a single-QTL model explains ∼20% of the phenotypic variance.

To extend the analysis further, I first performed a two-dimensional, two-QTL scan. For this analysis, I obtained several LOD scores under three models (Broman and Sen 2009). First, the full model ($M_{f}$) is the LOD comparing the two-QTL model to the null model of no QTL, while the second full model ($M_{fv1}$) compares the LOD of a two-QTL model to that of a single-QTL model. Notably, the full model allows for epistasis between the two QTL. Second, the additive models ($M_{a}$ and $M_{av1}$) are configured similarly to the full model, except no epistasis is allowed between QTL. Finally, a LOD score for epistasis is computed by subtracting the additive model from the full model (*i.e.*, $M_{i}=M_{f}-M_{a}$). Table 1 shows the two-locus combinations with significant LOD scores and their estimated physical and genetic locations.

**Table 1:** Two-QTL analysis output for the full, additive, and epistatic models

		Full Model				Additive Model				Epistasis
QTL1 Chr.	QTL2 Chr.	QTL1 Pos., cM, Mb	QTL2 Pos., cM, Mb	${\text{LOD}}_{f}$(*p* Value)	${\text{LOD}}_{fv1}$(*p* Value)	QTL1 Pos., cM, Mb	QTL2 Pos., cM, Mb	${\text{LOD}}_{a}$(*p* Value)	${\text{LOD}}_{av1}$(*p* Value)	${\text{LOD}}_{i}$(*p* Value)
2	5	84, 22.3	126, 16	93 (0)	42.83 (0)	82, 21.7	126, 16	91 (0)	40.84 (0)	1.99 (0.1)
5	5	120, 14.4	142, 22.7	59.9 (0)	9.78 (0)	118, 13.9	142, 22.7	58.6 (0)	8.43 (0)	1.34 (0.3)

The first significant two-QTL output identifies the chromosome 2 and 5 QTL that were identified in the first, single-QTL scan (Table 1, top row). A test of epistasis between these two loci does not uncover a significant interaction (LOD = 1.99, *p* value = 0.1). The lack of evidence for epistatic interaction between these two QTL means the full and additive models are effectively equivalent. The other significant two-QTL output identifies two additional QTL on chromosome 5 at 120 and 142 cM, also with no strong evidence of epistasis (Table 1). These results suggest that the large QTL on chromosome 5 may be underpinned by several QTL (at least three, according the two-QTL scan).

To examine the possibility of multiple QTL on chromosome 5, I fit an MQM with the putative QTL identified in the previous steps. I first fit a model with each of the chromosome 5 QTL (three so far) and the chromosome 2 QTL, assuming no interaction between any of the QTL (y∼Q1+Q2+Q3+Q4). This model resulted in an improvement on the best two-QTL model (LOD increase from 93 to 103, explaining ∼23% of the variance compared with 20% for the two-QTL model). Given the current MQM model, I scanned for additional QTL on the two chromosomes, and uncovered a QTL at the position of the *pup* marker (60 cM) with a LOD score of ∼4. After adding the latter QTL to the MQM model, I performed a pair-wise test for epistasis between all QTL pairs, and uncovered a marginally significant interaction between the chromosome 2 QTL (Q1) and the centromeric QTL on chromosome 5 (Q5). The final MQM model with the maximal LOD score and percentage variance explained is: y∼Q1+Q2+Q3+Q4+Q5+Q1:Q5. The full model result is shown in Table S2.

The evidence for multiple QTL on chromosome 5 is highly supported by the analyses so far. The LOD score for each individual QTL on that chromosome, however, is quite low (Table S3). This MQM model results in marginally significant LOD scores for three of the four chromosome 5 QTL, and each individually, explaining 0.3–24% of the phenotypic variance. In addition, the QTL at the *pup* marker (Q2, 60 cM) has a negative effect on the phenotype (Table S4). This is likely an artifact of the recombinant selection, as only three individuals in the mapping population are homozygous for *D. americana* alleles at that locus (Figure S4). The remaining three QTL on that chromosome appear to act additively (Figure S4). The physical coordinates of the QTL identified are given in File S4.

The last QTL method I used to identify regions on chromosome 5 is CIM, where markers at putative QTL can be selected as covariates. Here, I used the R/qtl implementation of CIM, where the number of marker covariates are defined and then forward selected by LOD score rank. In other words, if one marker covariate is selected, that marker will be the one closest to the QTL with the largest effect on the phenotype. Alternatively, if two markers are selected, the first marker will be as in the latter case and the second marker will be closest to the QTL with the second largest effect on the phenotype, and so on.

**Figure 4:**
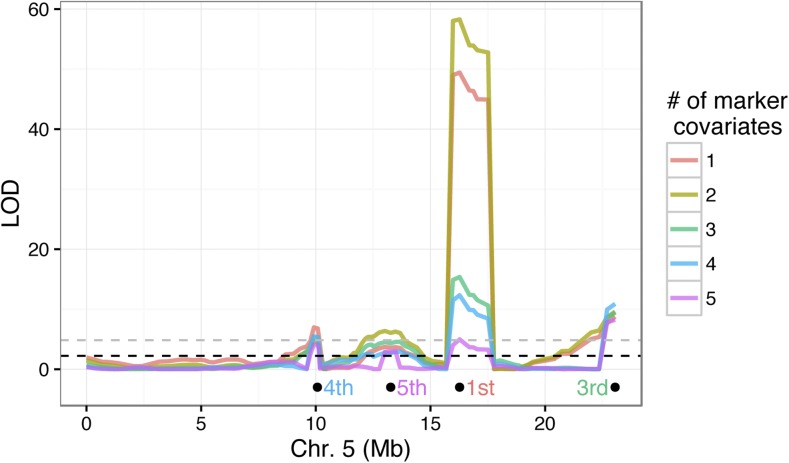
Composite interval mapping of QTL on chromosome 5. The LOD profiles are shown for each CIM run with a different number of marker covariates. The location of the *n*th marker selected in the analysis is shown below the LOD curve and the text color coded by analysis (the second marker is the chromosome 2 QTL). The dashed lines show the significance thresholds (α=0.05) for the CIM model using one covariate marker (black) and five covariate markers (gray).

I examined the LOD profile of CIM with up to five markers as covariates and found that, while the location of the QTL peaks are congruent between the MQM and CIM analyses, the magnitude of LOD scores at one of the QTL (Q4 at 127 cM, SSR116) varied dramatically depending on the number of covariates (Figure 4). This marker was identified in the single-QTL analysis as having the largest effect on the phenotype, and was also the marker with the highest LOD score in the analysis by Sweigart (2010b) of the paternal PMPZ phenotype. This QTL remains highly significant with up to four covariate markers, but the addition of the fifth marker (the adjacent Q3 at 116 cM) renders it nonsignificant.

#### RecIntLs:

I selected a set of recombinant individuals from the mapping population that had high fertilization success in the previous QTL analysis, repeatedly backcrossed their recombinant fifth chromosome (through nonrecombining males) into *D. americana*, and ultimately crossed siblings that are heterozygous for that chromosome to make the chromosome homozygous in a mostly *D. americana* background. This yielded a set of RecIntLs that can be subjected to further recombination for yet finer mapping. It is worth noting that these lines are not isogenic across the initially heterozygous portion of the fifth chromosome as homozygotes were selected based on the respective visible marker carried by the original recombinant father—new recombinants could have arisen when making the lines homozygous.

**Figure 5:**
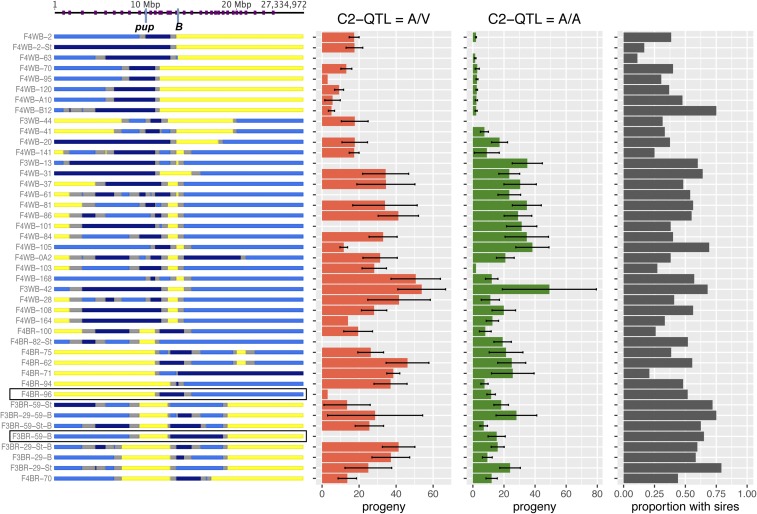
Recombinant introgression lines (RecIntLs). Chromosome 5 genotypes of RecIntLs are shown on the left (yellow = *D. americana* homozygote, dark blue = *D. virilis* homozygote, light blue = segregating *D. americana* and *D. virilis* alleles, gray = unknown). Purple ticks on the top indicate position of microsatellite markers on chromosome 5. The mean number (and SE) of progeny sired by individual males from each line (>20 per line) mated to *D. virilis* females is shown to the right [red bars = males heterozygous at chromosome 2 QTL (*va*), green bars = males homozygous for *D. americana* alleles at chromosome 2 QTL]. The black bars show the proportion of males that sired progeny for each RecIntL. These results show that the right half of chromosome 5 has an effect on PMPZ, whereas the left half of chromosome 5 contributes little or no effect on PMPZ. The two rectangles around the two RecIntL genotypes highlight the lines used in additional recombination mapping.

After generating the RecIntLs, I regenotyped the lines along the fifth chromosome at 36 markers to assess whether they were fixed for either *D. americana* or *D. virilis* alleles or contained heterozygous individuals. I then crossed individual males from each line (*n* ≥ 20 males per line) to assess whether regions on chromosome 5 that carry *D. virilis* alleles contain paternal loci capable of rescuing fertilization (Figure 5).

Four observations emerge from this approach. First, the regions that were initially heterozygous in the ancestral recombinant chromosome are largely heterogeneous within each RecIntL, *i.e.*, both *D. americana* and *D. virilis* alleles are segregating. Second, individuals within each RecIntL varied greatly in their fertilization abilities. Specifically, individuals that are heterozygous at putative QTL often do not produce progeny, suggesting that the mapped factors often show low penetrance. Third, one of the RecIntLs (F4BR-70, bottom of Figure 5) is heterozygous along a 2.5 Mb region near the center of the chromosome and has increased fertilization success that can sometimes be attributed to chromosome 5 alone (in cases where the chromosome 2 QTL genotype is A/A). This observation further narrows a key candidate region which coincides with Q3. Finally, although recombinant fifth chromosomes were introgressed for multiple generations, several RecIntLs are still segregating for *D. virilis* alleles on chromosome 2 (and possibly other chromosomes). Fortunately, several of the RecIntLs either do not carry or have a low frequency of *D. virilis* alleles on chromosome 2, as the effects of chromosome 2 on PMPZ might complicate efforts to fine-map factors on chromosome 5. These lines are thus especially valuable for further recombination-mapping on chromosome 5.

#### Further recombination and fine-mapping using RecIntLs:

I chose two of the RecIntLs, F3BR-59-B and F4BR-96 (highlighted in Figure 5), for further recombination mapping as they (1) are mostly homozygous for *D. americana* alleles on chromosome 2, and (2) carry the rescuing *D. virilis* alleles in the candidate QTL regions on chromosome 5. Importantly, both lines show sufficient fertilization rescue when crossed with *D. virilis* females.

To subject the fifth chromosomes of these two lines to further recombination, I crossed males from each line with *D. americana* females and allowed progeny to intercross for three generations. I then collected males homozygous for the visible marker (*B*) and crossed them individually with *D. americana* females to generate heterozygous sons carrying either the original recombinant fifth chromosome or a new recombinant (Figure S2). As before, these males were tested for fertilization ability by crossing with *D. virilis* females. Genotyping was carried out in two stages. First, only the fathers were genotyped to see whether the ancestral heterozygous region segregated for both *D. virilis* and *D. americana* alleles. Second, only subsets of sons sired by fathers segregating for both species’ alleles were genotyped. Both individual sons who sired many progeny and those who sired none were selected for genotyping.

**Figure 6:**
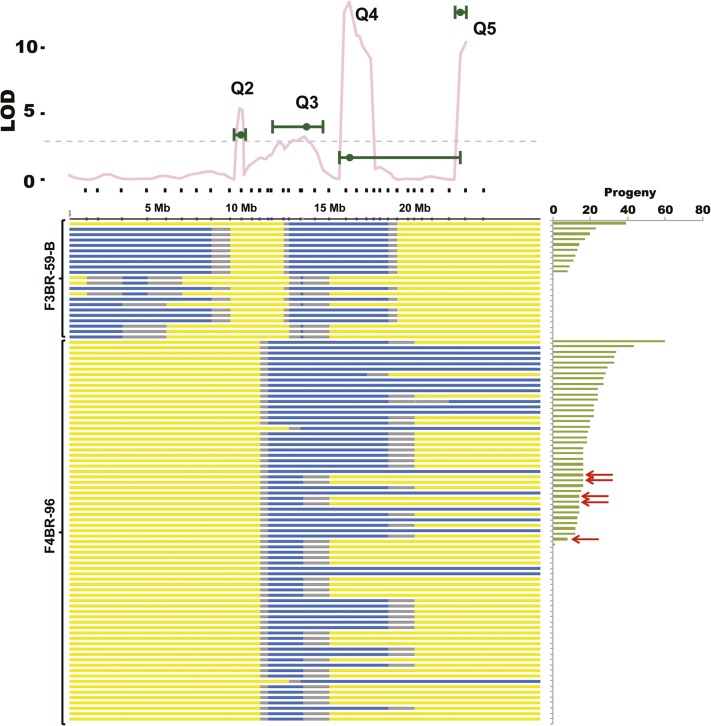
Genotypes of F3BR-59-B and F4BR-96 descendants after additional rounds of recombination. The LOD profile on the top is the CIM LOD profile where four markers at putative QTL are allowed to covary (from Figure 4). The green points on that plot are the LOD scores for each of the chromosome 5 QTL under the MQM model, and the error bars represent the estimated LOD interval (File S1). The genotype for each individual fifth chromosome is shown below the LOD plot and is indicated by color as described in Figure 2. Individual chromosomes are rank-ordered by number of progeny sired by each carrier male and are grouped by original RecIntL, with F3BR-59-B descendants shown at the top and F4BR-96 descendants below. The red arrows point to newly recombined F4BR-96 descendants that are heterozygous around the Q3 QTL region and have high fertilization success.

Ultimately, only a small number of fathers/sons fulfilled the criteria above. The genotypes and phenotypes of these sons are shown in Figure 6. As before, the region under Q4 (near SSR-116) on chromosome 5 has a large effect on fertilization success of recombinant males. Among F3BR-59-B descendants, only nonrecombined individuals sire progeny (top 10 chromosomes in Figure 6), while those that do not sire progeny include both nonrecombined and newly recombined males. The overlap between the MQM LOD intervals (green error bars in the top portion of Figure 6), the CIM LOD profile (pink line in the top portion of Figure 6) and the heterozygous regions among F3BR-59-B recombinants suggests that Q4 (SSR116) indeed contributes to paternal PMPZ, and at least one causal locus lies in a region that spans 3.3 Mb. The adjacent causal locus under Q3 may lie directly to the left of Q4 (within 4.5 Mb), or may lie to the left of the heterozygous region.

Among F4BR-96 descendants, the regions under Q3, Q4, and Q5 clearly contribute to fertilization success. Unlike Q4 in the previous analysis, no recombinants are recovered that are heterozygous in the Q5 regions to the exclusion of other QTL on that chromosome (Figure 6). However, some newly recombined individuals are heterozygous almost exclusively around the Q3 QTL (∼4 Mb). Few of these individuals maintain modest fertilization success when crossed with *D. virilis* females. Specifically, five F4BR-96 descendants lack *D. virilis* alleles at Q4 and Q5, yet are able to sire 8–16 progeny (red arrows in Figure 6). This confirms that the Q3 factor is likely independent of (and lies to the left of) the major Q4 QTL.

Several conclusions emerge from the mapping approaches described here. First, at least three paternal loci reside on chromosome 5. At least two of these reside near the center of the chromosome and (one or both) have the largest effect on fertilization success. Second, the recombinants that carry the smallest heterozygous region on chromosome 5 and that have increased fertilization success relative to pure *D. americana* show relatively low rescue compared to recombinants that contain larger heterozygous regions on that chromosome. This latter observation suggests that paternal loci on chromosome 5 have cumulative, and perhaps roughly additive, effects on PMPZ.

## Discussion

Members of the *virilis* group of *Drosophila* are strongly isolated by PMPZ barriers; postzygotic barriers, on the other hand, are less common. Despite this asymmetry, a number of previous studies have focused on the genetics of postzygotic isolation in this group (Orr and Coyne 1989; Heikkinen and Lumme 1998; Sweigart 2010a), while the genetic study of PMPZ has, by comparison, been somewhat neglected (but see Sweigart 2010b). Furthermore, the biological mechanisms underlying PMPZ in this species group have rarely been studied (Patterson and Stone 1949), and remain poorly understood.

PMPZ phenotypes in *Drosophila* might have several causes. For example, interspecific sperm length differences may contribute to inefficient storage, especially in the seminal receptacle. Because sperm length and seminal receptacle length can coevolve within *Drosophila* species, closely related species may become gametically incompatible as a result of sperm length differences (Miller *et al.* 2003; Miller and Pitnick 2002, 2003; Manier *et al.* 2013a,b). In addition, sperm surface proteins that are likely involved in the sperm’s interaction with the female reproductive tract or the egg micropile might diverge rapidly between species (Pitnick *et al.* 2009; Karr and Dorus 2012), rendering heterospecific sperm less efficient at fertilizing eggs from another species. Finally, because sperm storage in *Drosophila* is facilitated by seminal fluid proteins that are transferred in the male ejaculate (Tram and Wolfner 1999), sperm storage defects in heterospecific inseminations may be caused by divergent seminal fluid proteins. For example, in *D. melanogaster*, one particular seminal fluid protein (Acp36DE) is required for normal sperm storage, may influence maintenance of sperm in the female storage organs, and may play a role in sperm competition (Clark *et al.* 1995; Neubaum and Wolfner 1999; Qazi and Wolfner 2003).

Here, I have shown that the PMPZ incompatibility between *D. virilis* females and *D. americana* males involves loss of *D. americana* sperm after successful storage in the spermathecae and the seminal receptacle of *D. virilis* females. Loss from the seminal receptacle occurs faster than loss from the spermathecae. In some *Drosophila* species (*e.g.*, *D. melanogaster*), the seminal receptacle is considered the primary sperm storage organ and the main source of fertilizing sperm (Pitnick *et al.* 1999), but in other species (*e.g.*, *D. simulans* and *D. mauritiana*) the spermathecae appear to act as the primary sperm storage organ (Manier *et al.* 2013a,b).

I have also shown that several loci (at least four) contribute to the paternal side of the incompatibility between *D. americana* males and *D. virilis* females. One of these loci resides on (or near) an inverted region on chromosome 2, and is therefore essentially unmappable, at least by recombination studies. Chromosome 5 has the largest effect on the paternal side of PMPZ isolation, and fortunately recombines between *D. americana* and *D. virilis*. Using a series of recombination-mapping approaches, I identified three regions on chromosome 5 that have a large effect on the paternal side of the incompatibility and appear to act roughly additively. Other modifiers across the genome likely contribute to fertilization success between species. The RecIntLs generally show much reduced fertilization success relative to the backcross individuals used in the QTL analysis. The genetic background of the introgression lines is largely *D. americana* material, but the backcross individuals used in the QTL analyses segregate for several *D. virilis* alleles across the genome. Thus the paternal side of the PMPZ incompatibility between *D. virilis* females and *D. americana* males has a somewhat complex genetic basis, with at least four factors having a large effect on the phenotype. As some (unknown) number of loci must contribute to the female (*D. virilis*) side of this PMPZ incompatibility, the total number of loci involved in this barrier must be considerable.

The processes affected in the PMPZ isolation between *D. americana* and *D. virilis* (*i.e.*, sperm transfer, storage, and/or viability, *etc*.) are generally thought to be modulated by accessory gland secretions transferred in the male ejaculate (Wolfner 2009). Accessory gland proteins (Acps) are well characterized in *D. melanogaster*, and have been implicated in several postcopulatory events that facilitate reproduction (Avila *et al.* 2011). Furthermore, genetic variation in Acps has been associated with several sperm competition phenotypes in *D. melanogaster* (Fiumera *et al.* 2005, 2007). Acps, however, are not well characterized in the *virilis* group, and given their rapid evolution, many Acps in *D. melanogaster* likely have few homologs among distantly related *Drosophila* species (Kelleher *et al.* 2009).

The analyses presented here represent progress in both the phenotypic and genetic study of PMPZ in *Drosophila*. Further investigations of the genetic basis of this incompatibility could greatly benefit from the identification of reproductive genes that facilitate postcopulatory processes within the female reproductive tract.

## Supplementary Material

Supplemental Material
