# Supplementary material for: Multiple Genes Cause Postmating Prezygotic Reproductive Isolation in the Drosophila virilis Group
Source: G3 (Bethesda). 2016 Oct 10;6(12):4067–76. doi: 10.1534/g3.116.033340 (PMC5144975; doi:10.1534/g3.116.033340)
Supplement: Supplemental Material [file supp_6_12_4067__index.html]

Multiple Genes Cause Postmating Prezygotic Reproductive Isolation in the Drosophila virilis Group — Supplemental Material 

# Multiple Genes Cause Postmating Prezygotic Reproductive Isolation in the *Drosophila virilis* Group

## Supplemental Material for Ahmed-Braimah, 2016

**Files in this Data Supplement:**

- Figure S1 - Recombination rates for markers on chromosome 5. (.pdf, 160 KB)
- Figure S2 - Crossing scheme to obtain additional recombinants from RecIntLs. (.pdf, 151 KB)
- Figure S3 - Single-QTL scan results. (.pdf, 216 KB)
- Figure S4 - Phenotypic effects of adjacent QTL. (.pdf, 300 KB)
- Table S1 - Genetic markers used in this study, their coordinates, and primer sequences. (.pdf, 168 KB)
- Table S2 - Full MQM model result. (.pdf, 151 KB)
- Table S3 - Drop one QTL at a time ANOVA. (.pdf, 149 KB)
- Table S4 - Estimated phenotypic effects and physical location of putative QTL. (.pdf, 140 KB)
- File S1 - This file contains the R code used to perform the QTL analysis. (.zip, 5 KB)
- File S2 - This file contains the genotype and phenotype data and the genetic map information. (.csv, 209 KB)
- File S3 - This file describes the correspondence of genetic distances to physical distances. (.zip, 2 KB)
- File S4 - This file contains output from the statistical analysis in File S1 that was used to produce part of Figure 6. (.zip, 1 KB)
- File S5 - Movie of *D. virilis* female reproductive tract 24 hours after conspecific copulation. Structural components of the reproductive tract are described in Figure 1. Rampant sperm movement can be observed throughout the seminal receptacle. (.zip, 8.48 MB)
- File S6 - Movie of *D. virilis* female reproductive tract 24 hours after heterospecific copulation. This is a magnified portion of the seminal receptacle shown in Figure 1A (right), where only one motile sperm is found. (.zip, 15.9 MB)
