# Supplementary material for: Multiple Genes Cause Postmating Prezygotic Reproductive Isolation in the Drosophila virilis Group
Source: G3 (Bethesda). 2016 Oct 10;6(12):4067–76. doi: 10.1534/g3.116.033340 (PMC5144975; doi:10.1534/g3.116.033340)
Supplement: Supplemental Material [file supp_g3.116.033340_FigureS1.pdf]

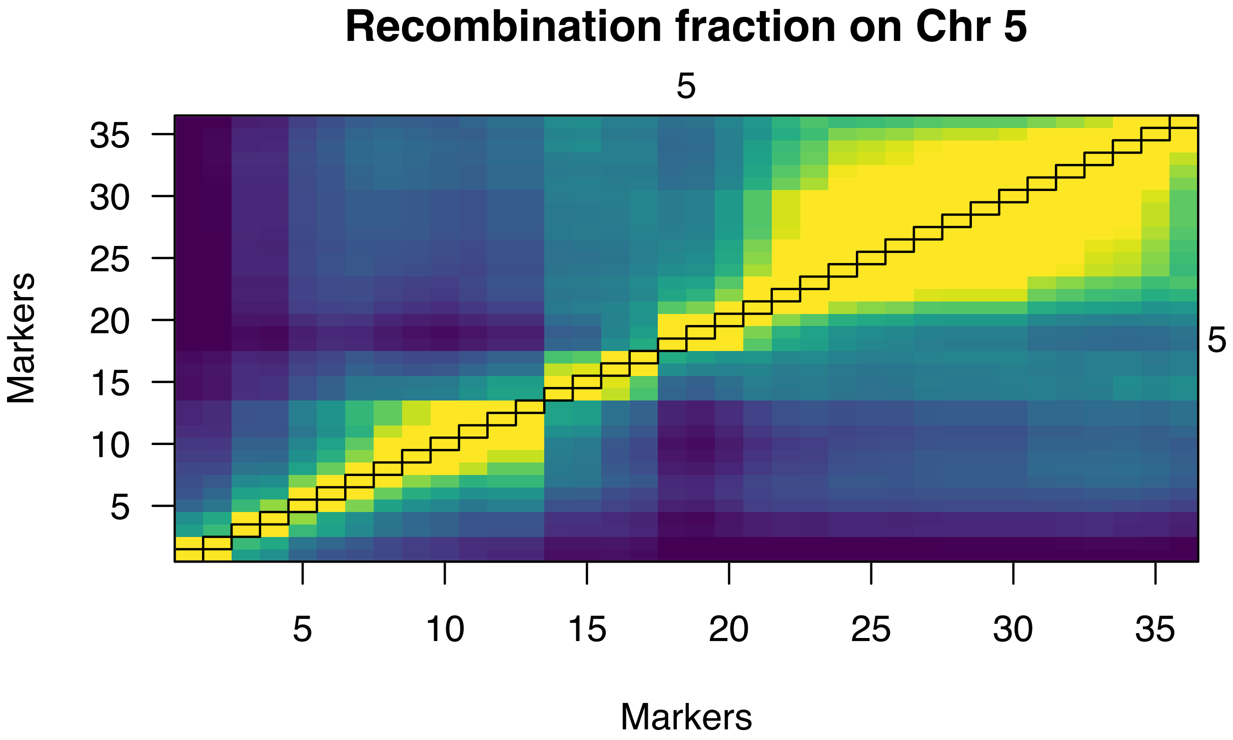

Figure S1 Recombination rates for markers on chromosome 5. Yellow squares depict low rates of low recombination (i.e., tight linkage), and dark blue squares depict higher recombination. Note the high recombination between marker 13 (*pup*) and marker 19 (*B*), between which recombinants were selected. The recombinant selection has the consequence of reducing recombination to the right of *B* (there is also a higher marker density in this region) and to the left of *pup*.
