# Supplementary material for: Multiple Genes Cause Postmating Prezygotic Reproductive Isolation in the Drosophila virilis Group
Source: G3 (Bethesda). 2016 Oct 10;6(12):4067–76. doi: 10.1534/g3.116.033340 (PMC5144975; doi:10.1534/g3.116.033340)
Supplement: Supplemental Material [file supp_g3.116.033340_FigureS3.pdf]

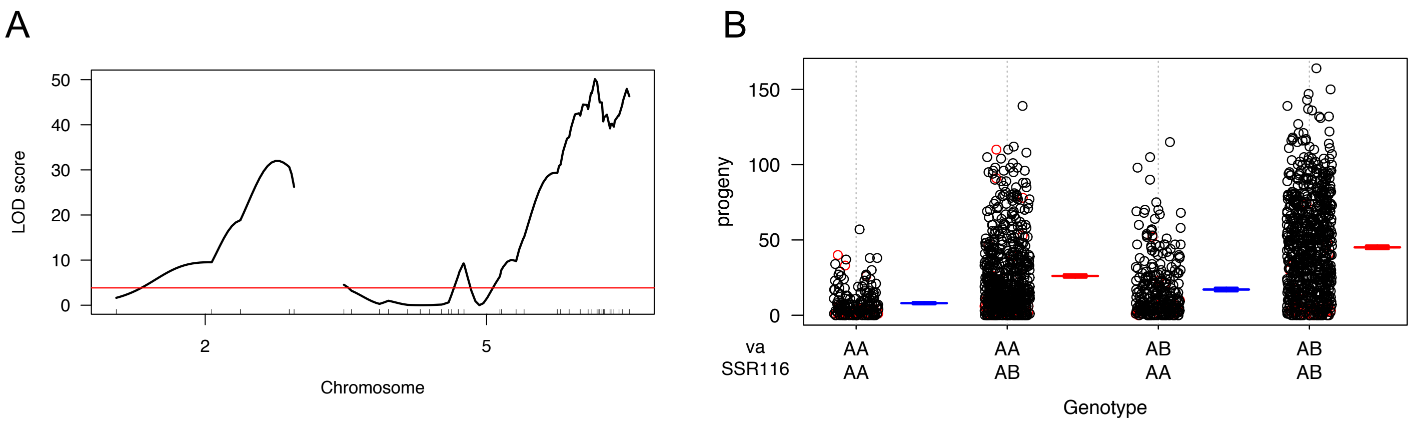

Figure S3 Single-QTL scan results. (A) The LOD profile of the single-QTL scan on chromosomes 2 and 5. The significance threshold ($\alpha = 0.01$) is shown in red. (B) The phenotypic effects of the markers with the highest LOD scores on chromosome 2 (*va*) and on chromosome 5 (SSR-116) partitioned by genotype at both markers. Each circle represents an individual in the mapping population (a red circle indicates a missing/imputed genotype for one of the two markers, which is *va* in all cases), and the average effect of the two genotype combinations is indicated in red or blue (error bars indicate standard error).
