# Supplementary material for: Multiple Genes Cause Postmating Prezygotic Reproductive Isolation in the Drosophila virilis Group
Source: G3 (Bethesda). 2016 Oct 10;6(12):4067–76. doi: 10.1534/g3.116.033340 (PMC5144975; doi:10.1534/g3.116.033340)
Supplement: Supplemental Material [file supp_g3.116.033340_FigureS4.pdf]

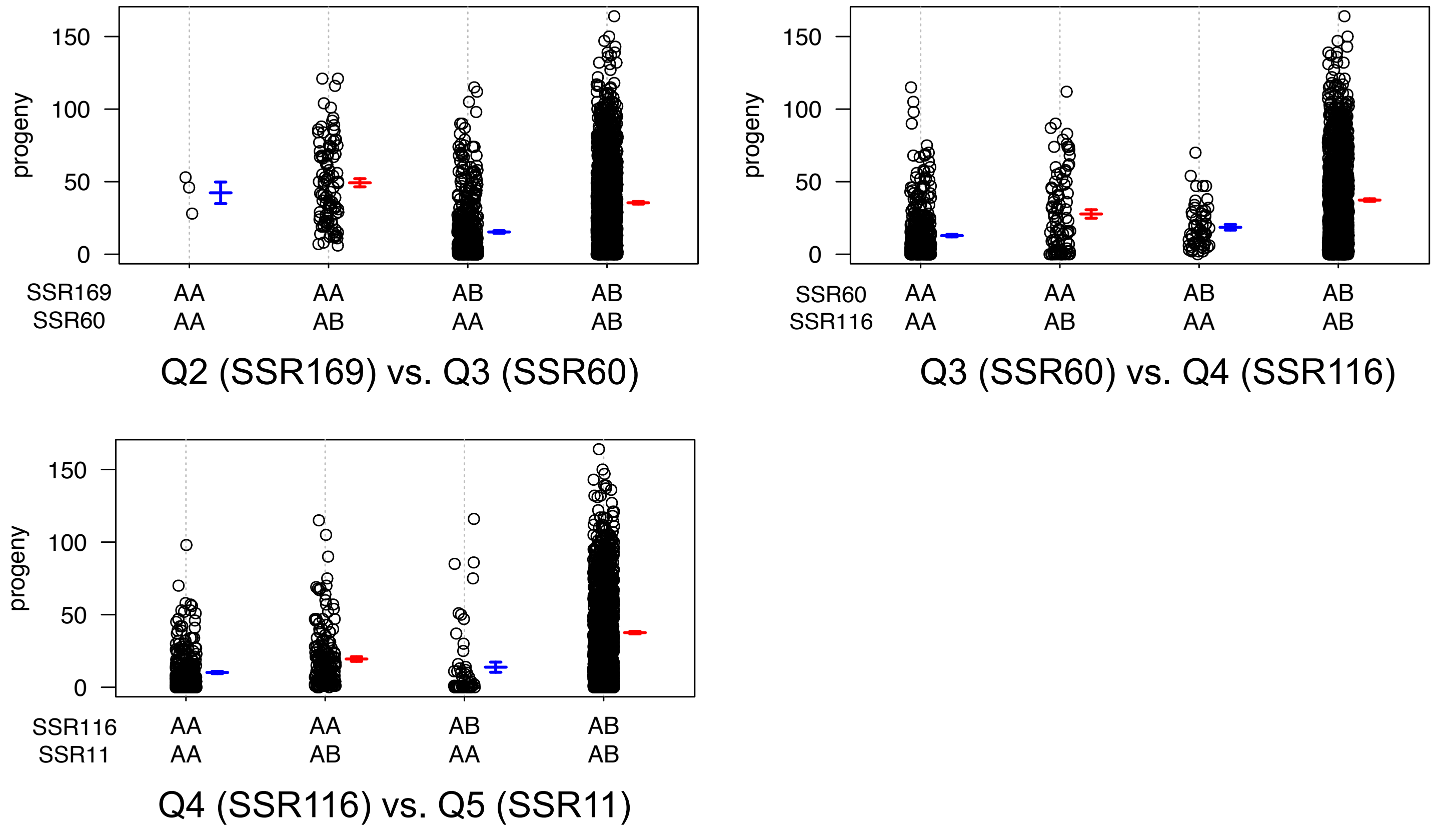

Figure S4 Phenotypic effects of adjacent QTL. The phenotypic effect of QTL on chromosome 5 is plotted for each adjacent pair (microsatellite marker name in parentheses). Each circle represents the progeny count for a given pairs of genotype combinations at the two markers, where "A" is the *D. americana* allele and "B" is the *D. virilis* allele. The mean phenotypic effect and standard error for each genotype combination is shown in red or blue.
