# Supplementary material for: Multiple Genes Cause Postmating Prezygotic Reproductive Isolation in the Drosophila virilis Group
Source: G3 (Bethesda). 2016 Oct 10;6(12):4067–76. doi: 10.1534/g3.116.033340 (PMC5144975; doi:10.1534/g3.116.033340)
Supplement: Supplemental Material [file supp_g3.116.033340_TableS1.pdf]

**Table S1** Genetic markers used in this study, their coordinates, and primer sequences.

| Marker | Scafffold[1] | Scaffold coords. | Chr. | Chr. coords.[2] | Forward | Reverse |
|---|---|---|---|---|---|---|
| **SSR72** | 12822 | 1724188-1724354 | 2 | 1724188-1724354 | TGCAGTCAAAACTGGGTCAA | GCAAGACCAAAAGTGCGAGT |
| **SSR33** | 13047 | 16959223-16959455 | 2 | 6359965-6360197 | CATTTCCTGCTGGCTAGCTT | GTCAGACACAGCGACGACAT |
| **SSR74** | 13047 | 14987288-14987501 | 2 | 8331938-8332112 | CCTTGGCATGTTTTAGAGCAA | AAGCGACGCGCAAAATATAC |
| ***va*** | 12855 | N/A | 2 | N/A | N/A | N/A |
| **SSR81** | 12855 | 9909979-9910129 | 2 | 33229398-33229548 | TGCACGTAAGTGTGAATCTGC | GTGCCAATCACATCGCAGT |
| **SSR66** | 12823 | 997910-998114 | 5 | 997910-998118 | CTCGCTGTCGCAATGTTTAC | GCCGCAAATAAAATGGTGAT |
| **SSR104** | 12823 | 1689490-1689676 | 5 | 1689490-1689676 | CGTCACGTACCGAGAAGACA | AGTACGTGTGGCATCAGCAA |
| **SSR121** | 10324 | 594567-594746 | 5 | 3069112-3069291 | TGCGCACTAAATGAGATGCT | CAAAGGCGTCATGGTAACCT |
| **SSR123** | 12875[3] | 912756-912964 | 5 | 4530387-4530596 | AAGGTTTCCAGCGAATAGGC | CGTTGCTTACGTCAATCGAG |
| **SSR119** | 12875 | 20444191-20444368 | 5 | 5610567-5610743 | GAGAAGCGAGAGGCACACTT | CCAGGTAGGCTCTGTCCAAC |
| **SSR58** | 12875 | 19509634-19509842 | 5 | 6545087-6545300 | TGCCTAGCATTTGGCACTTA | AAAAGAGCGTGGCAAAGAAA |
| **SSR192** | 12875 | 18634421-18634630 | 5 | 7420304-7420513 | ATCAAGACCCAAGCCGGAAACCAG | CTGGTTTCCGGCTTGGGTCTTGAT |
| **SSR190** | 12875 | 17808454-17808680 | 5 | 8246254-8246480 | TGCCTGCTGATGAATCACGTGAC | TGTCACGTGATTCATCAGCAGGCA |
| **SSR95** | 12875 | 16718920-16719133 | 5 | 9335798-9336014 | TGTGCCTGCTGACAAAACAT | ACACTGCCTGCTTGCATTTA |
| **SSR138** | 12875 | 16037240-16037447 | 5 | 10017486-10017694 | TCGTACCAATGTTGTCAATGC | ACAGGAAGTGGGTGGAAGTG |
| **SSR155** | 12875 | 15450044-15450076 | 5 | 10604771-10604976 | TGGGACAAATGTGCCAAATA | ATATGCTCGGTCGGAGATTC |
| **SSR169** | 12875 | 14987033-14987196 | 5 | 11067738-11067901 | CATCAAGGAGCTGGCCTATC | ACTTCTCCGCCGTACAACTG |
| ***pup*** | 12875 | 14961086-14964626 | 5 | 11084412-11091072 | N/A | N/A |
| **SSR181** | 12875 | 14501333-14501602 | 5 | 11553332-11553601 | CAAGGGCATTGACTTCTACG | GGTTTTGGCTCAAAATCCAG |
| **SSR23** | 12875 | 14309322-14309540 | 5 | 11745404-11745612 | AAACTGGCAGATGGGCATAG | CCACGATTTCAGAAGCACAA |
| **SSR179** | 12875 | 13607115-13607443 | 5 | 12447491-12447819 | TGACGCAGTTGGCAATTAAC | ACTTGCCGATGAAGTTTTCG |
| **SSR178** | 12875 | 13299025-13299315 | 5 | 12755619-12755909 | AGTGCCACAAAACCGAAAAC | GAATGCCGCATAGATTCCTC |
| **SSR93** | 12875 | 12612982-12613197 | 5 | 13441731-13441952 | TCGAACTCGATCTTCTAGCTTCT | TTGAACTCTTCACGCGACTTT |
| ***B*** | 12875 | 12492243-12542471 | 5 | 13512463-13562691 | N/A | N/A |
| **SSR188** | 12875 | 11790642-11790879 | 5 | 14264055-14264292 | CGACGCACACAAACGCAATACTTTG | CAAAGTATTGCGTTTGTGTGCGTCG |
| **SSR60** | 12875 | 10970098-10970252 | 5 | 15084664-15084836 | CAAAAGTGTTGCCTTGATGG | GGGTTCTAGCCCCCAAATAA |
| **SSR90** | 12875 | 9976996-9977241 | 5 | 16077689-16077938 | ACTTTGCCAAGCTGTGAAGG | GCGTCTCGTATGCTCTGCTA |
| **SSR176** | 12875 | 9364934-9365179 | 5 | 16689755-16690000 | TGCCATTTGCGTATTTTGTG | CTGCAATAGGGGGTCAAATAG |
| **SSR116** | 12875 | 8785474-8785673 | 5 | 17269263-17269460 | CCCCATTGAAAGTTCATCCA | GTCAGGAGGCCACATTGTTT |
| **SSR118** | 12875 | 8359663-8359861 | 5 | 17695072-17695271 | GCCCAAAATTCTTAGCCAAA | TGGCTTGGGTACTGGTTTCT |
| **SSR88** | 12875 | 8014665-8014816 | 5 | 18040111-18040269 | CCAAAAGGCAGGACCATAAA | TTGCGTAGACACCACAAGGT |
| **SSR124** | 12875 | 7542114-7542326 | 5 | 18512607-18512820 | CGCTTAAACGATCCAACGAT | GTTCATAACCGGTGCTCGAT |
| **SSR125** | 12875 | 7024865-7025043 | 5 | 19029891-19030069 | AGCGCTGATGCTTAAACTGC | TTACTTGCTGACCCACTTACG |
| **SSR87** | 12875 | 7014393-7014574 | 5 | 19040357-19040541 | CAGCGCGTGCTGATTAGTTA | TACAGCTGGCTGCGTTTATG |
| **SSR126** | 12875 | 6381246-6381454 | 5 | 19673479-19673688 | AATTGCCAAAGAAACCACCA | AGCGGCTCGTCGTCTGTA |
| **SSR129** | 12875 | 6028577-6028753 | 5 | 20026180-20026357 | ACCATGAGCAGGCATCAGA | CCGGCAATCGTTCTTTAACT |
| **SSR133** | 12875 | 5581289-5581484 | 5 | 20473430-20473645 | CTCGCAACTTGGCAGACATA | TGCCCCAATGTAACTGCTATC |
| **SSR62** | 12875 | 4985233-4985531 | 5 | 21069407-21069701 | TGTTAGTTGGCAGCGCAAT | GATTATGCGTGTTGCAGTCG |
| **SSR183** | 12875 | 4001657-4001814 | 5 | 22053120-22053277 | CGTATTTGAGTGCAACGCGTTTG | CCAAACGCGTTGCACTCAAATACG |
| **SSR13** | 12875 | 3052296-3052513 | 5 | 23002420-23002638 | CTTTATCCCGATGTGCGAGT | GCCGCAATAAACTGGAAAAG |
| **SSR11** | 12875 | 2002257-2002505 | 5 | 24052429-24052677 | TTGGCAGAGCTTTCTCACCT | CTAAACGGGCCTCCACATT |

[1] FlyBase genome release dvir1.2.
[2] Chromosome coordinates based on arrangement of genome scaffolds on Muller elements (Schaeffer *et al.* 2008; Ahmed-Braimah and Sweigart 2015).
[3] Misplaced scaffold portion in original genome assembly (Schaeffer *et al.* 2008).
