# Supplementary material for: Multiple Genes Cause Postmating Prezygotic Reproductive Isolation in the Drosophila virilis Group
Source: G3 (Bethesda). 2016 Oct 10;6(12):4067–76. doi: 10.1534/g3.116.033340 (PMC5144975; doi:10.1534/g3.116.033340)
Supplement: Supplemental Material [file supp_g3.116.033340_TableS2.pdf]

Table S2 Full MQM model result

	df	SS	MS	LOD	%var	*p-value*(χ^2)	*p-value*(F)
**Model**	6	397771.9	66295.31	111.51	24.33	0	0
**Error**	1835	1237202.8	674.23				
**Total**	1841	1634974.7					
