# Supplementary material for: Multiple Genes Cause Postmating Prezygotic Reproductive Isolation in the Drosophila virilis Group
Source: G3 (Bethesda). 2016 Oct 10;6(12):4067–76. doi: 10.1534/g3.116.033340 (PMC5144975; doi:10.1534/g3.116.033340)
Supplement: Supplemental Material [file supp_g3.116.033340_TableS3.pdf]

Table S3 Drop one QTL at a time ANOVA

QTL	df	LOD	%var	F value	*p-value*(F)
**2@84.0 (*va*)**	2	44.6	8.9	108.1	< 2e-16
**5@60.3 (SSR169)**	1	3.4	0.6	15.6	8.14e-05
**5@116.0 (SSR60)**	1	4.0	0.8	18.4	1.84e-05
**5@127.1 (SSR116)**	1	1.7	0.3	7.6	0.005720
**5@142.0 (SSR11)**	2	12.7	2.4	29.6	2.30e-13
**2@84.0:5@142.0**	1	3.1	0.6	14.5	0.000148
