# Supplementary material for: Multiple Genes Cause Postmating Prezygotic Reproductive Isolation in the Drosophila virilis Group
Source: G3 (Bethesda). 2016 Oct 10;6(12):4067–76. doi: 10.1534/g3.116.033340 (PMC5144975; doi:10.1534/g3.116.033340)
Supplement: Supplemental Material [file supp_g3.116.033340_TableS4.pdf]

Table S4 Estimated phenotypic effects and physical location of putative QTL

	Estimated effect	S.E.
**Intercept**	26.7	1.5
**2@84.0 (va)**	15.2	1.8
**5@60.3 (SSR169)**	-10.5	2.7
**5@116.0 (SSR60)**	9.2	2.2
**5@127.1 (SSR116)**	6.9	2.5
**5@142.0 (SSR11)**	13.7	2.2
**2@84.0:5@142.0**	13.6	3.6
